# Implementation of Wearable Sensors and Digital Alerting Systems in Secondary Care: Protocol for a Real-World Prospective Study Evaluating Clinical Outcomes

**DOI:** 10.2196/26240

**Published:** 2021-05-04

**Authors:** Fahad Mujtaba Iqbal, Meera Joshi, Sadia Khan, Hutan Ashrafian, Ara Darzi

**Affiliations:** 1 Division of Surgery and Cancer Imperial College London London United Kingdom; 2 West Middlesex University Hospital London United Kingdom

**Keywords:** remote sensing technology, clinical trial, patient deterioration, monitoring, ambulatory, wearable

## Abstract

**Background:**

Advancements in wearable sensors have caused a resurgence in their use, particularly because their miniaturization offers ambulatory advantages while performing continuous vital sign monitoring. Digital alerts can be generated following early recognition of clinical deterioration through breaches of set parameter thresholds, permitting earlier intervention. However, a systematic real-world evaluation of these alerting systems has yet to be conducted, and their efficacy remains unknown.

**Objective:**

The aim of this study is to implement wearable sensors and digital alerting systems in acute general wards to evaluate the resultant clinical outcomes.

**Methods:**

Participants on acute general wards will be screened and recruited into a trial with a pre-post implementation design. In the preimplementation phase, the SensiumVitals monitoring system, which continuously measures temperature, heart, and respiratory rates, will be used for monitoring alongside usual care. In the postimplementation phase, alerts will be generated from the SensiumVitals system when pre-established thresholds for vital parameters have been crossed, requiring acknowledgement from health care staff; subsequent clinical outcomes will be analyzed.

**Results:**

Enrolment is currently underway, having started in September 2017, and is anticipated to end shortly. Data analysis is expected to be completed in 2021.

**Conclusions:**

This study will offer insight into the implementation of digital health technologies within a health care trust and aims to describe the effectiveness of wearable sensors for ambulatory continuous monitoring and digital alerts on clinical outcomes in acute general ward settings.

**Trial Registration:**

ClinicalTrials.gov NCT04638738; https://clinicaltrials.gov/ct2/show/NCT04638738.

**International Registered Report Identifier (IRRID):**

DERR1-10.2196/26240

## Introduction

Vital signs and their trends are crucial in recognizing clinical deterioration, with changes often occurring several hours prior to an adverse event [1-7]. Consequently, monitoring of these physiological parameters forms a fundamental component of providing effective clinical care. Despite this, patient deterioration can go undetected, resulting in adverse clinical outcomes such as late referrals to intensive care units, increased morbidity, and mortality [8-13].

Routinely measured vital signs include heart rate, respiratory rate, temperature, blood pressure, oxygen saturation (and supplemental oxygen), and level of consciousness. Individuals admitted to nonintensive (general) hospital wards undergo intermittent monitoring of these vital signs. A “track and trigger” early warning score (EWS) is recommended in the United Kingdom; the National Early Warning Score 2 (NEWS2) provides guidance on escalation protocols and the monitoring frequency of vital signs. Each vital parameter is individually scored according to severity and combined for a total NEWS score. Most frequently, observations are performed every 4-6 hours unless the patient is acutely unwell [14].

EWSs are predicated around the idea that hospital inpatients at high risk of deterioration are identified early through prodromal vital parameter alterations (eg, elevated respiratory rate), enabling early intervention [15]. Since their implementation, EWSs have shown good predictive value for deterioration and have improved clinical outcomes [16]. However, their intermittent nature is a critical limitation that enables acute deterioration between measurements to be easily missed [16].

Wearable sensors and digital alerting mechanisms offer a potential solution to this issue. Continuous monitoring through novel wearable sensors provides near real-time monitoring of vital signs without hindering ambulation. It is theorized that continuous monitoring will enable earlier detection of deterioration through a culmination of additional data points and reduced reliance on availability of medical personnel for performing observations [17]. Alerts are subsequently generated, informing health care professionals when pre-established thresholds for vital parameters, often tailorable, are breached. However, the evidence supporting the use of wearable sensors outside of intensive care settings remains mixed and limited [18,19]. The heterogeneity of study designs and complexity of interventions used, however, limit meaningful conclusions.

Here, we describe the design of our trial, in which we implement wearable sensors and digital alerting systems in acute general wards in a National Health Service hospital; we also describe the implementation strategy and the evaluation of the resultant clinical outcomes (eg, length of stay, mortality, need for intensive care).

## Methods

### Study Design

This is a single-center, pre-post design in which digital alerting systems are implemented on acute wards. The preimplementation phase dated from September 2017 to August 2019, and the postimplementation phase is currently underway.

The preimplementation phase involved using the SensiumVitals system in accordance with usual care. However, health care staff were not able to view the continuous vital sign monitoring data or the digital alerts generated for abnormal parameters. Usual care, in our institution, involves intermittent monitoring of vital signs in accordance with NEWS2.

In the postimplementation phase, alerting systems following recognition of abnormal parameters will be included. These alerts will be transmitted to mobile devices and central monitoring hubs, with alert acknowledgement required from health care staff.

All participants provided informed consent. Ethical approval for this study has been granted by a Research Ethics Committee (Integrated Research Application System: 222979). The trial will be performed in accordance with Good Clinical Practice guidelines and the Declaration of Helsinki. Patient data will be anonymized to ensure privacy. Storage and handling of personal data will comply with the General Data Protection Regulation.

### Intervention Protocol for Alerts

All alerts will be viewed by dedicated trained nursing staff covering the wards. Alerts will be generated when measured vital signs exceed pre-established thresholds for 10 consecutive minutes. These thresholds can be individually tailored but are initially programmed to trigger if the patient’s temperature exceeds 38.1 ºC, respiratory rate is over 25 breaths per minute, and heart rate is over 131 beats per minute, in accordance with NEWS2 cutoffs [14]. All incoming alerts are deemed to be of potential clinical relevance, and potential outcomes include but are not limited to reviewing the individual, repeating observations, increased frequency of monitoring, and escalation to a responsible physician.

### Stakeholder Engagement

Several stakeholders will continue to be engaged during the establishment of the project; permission from the Estates and Information Technology departments has been obtained. This permission ensured that bridges were installed by the hospital Estates department, enabling adherence with local policies. Monitoring software has been integrated with the hospital admissions data system, enabling consenting participants to be easily added to the system. Data will be stored and retained on hospital networks, alleviating data security concerns. Senior clinicians have been informed of the project through engagement meetings to drive recruitment and elucidate the aims of the study. Nursing staff have been trained directly to use the system; ad hoc refreshers will be available throughout the duration of the study.

### Eligibility Criteria

Adults (aged over 18 years) admitted to general wards who are able to understand the participant information sheet are eligible for inclusion. Individuals with cardiac implantable electronic devices, who experience a skin reaction to the wearable sensor or its components, who have an open chest wound, or who withdraw consent will be excluded.

Recruitment will be aided by the responsible clinical team, who will deem individuals suitable to participate.

### Data Collection

After enrolment, data will be routinely collected by two research nurses and a clinical fellow. Outcomes will be obtained from case note review, SensiumVitals data, and electronic health records, enabling prospective data collection.

### Outcome Measures

Outcome measures will include hospital length of stay; critical NEWS (defined as 7 or over); number of admissions to intensive care; mortality; sepsis events; and time to antibiotics.

To understand the acceptability and usability of the SensiumVitals system by participants and health care staff, mixed methods analysis (semistructured interviews and questionnaires) will be undertaken in the postimplementation phase. For participants, these interviews and questionnaires will be administered 24 hours after the SensiumVitals sensor has been applied, enabling familiarity with the sensor and maximizing of data capture before potential hospital discharge. Due to the nature of the shift patterns, a set time point has not been chosen for health care staff; however, the studies will only be conducted once familiarity with the system has been established. All key stakeholders will also be invited to take part in semistructured interviews to determine barriers and facilitators to implementing wearable sensors and alerting mechanisms within the hospital.

The questionnaires consist of 5-point Likert scale responses (strongly disagree to strongly agree), with elements adapted from the validated System Usability Scale [20].

Face-to-face interviews will be conducted by the lead researcher (FI) using a predetermined topic guide. Data collection will be an iterative process; emerging recurring concepts were incorporated into the interview guide for further exploration with remaining participants. Interviews will be recorded, anonymized, transcribed, and entered into NVivo 12 (QSR International) for analysis.

### Statistical Analysis

The Shapiro-Wilk test will be used to check variables for Gaussian distribution. Data sets will be presented as absolute numbers of patients with the respective percentage per group or as parameter mean and standard deviation or median and range, depending on distribution. Descriptive statistics will describe the baseline characteristics of the participants, alerting frequencies, and events.

For comparisons of interval-scaled variables between the two groups, 2-tailed, unpaired *t* tests will be performed. Nonparametric between-group testing will be undertaken with 2-tailed Mann-Whitney U tests. Additionally, the chi-square test or Fisher exact test will be applied to nominal scale data.

Propensity score matching will be performed for differences among baseline demographics between pre-post phases, reducing bias from confounding variables. Analyses will be performed in SPSS (IBM Corporation), Stata (StataCorp LLC), and GraphPad (GraphPad Software Inc).

Mixed methods analysis will be undertaken for the questionnaire and semistructured interview data. Frequency distributions will be generated for Likert scale responses. Interview transcripts will be analyzed using thematic analysis [21]. The results will be discussed until consensus is reached.

### Power Calculations

Formal power calculations are not possible on any of the outcomes, given the lack of surrounding data. However, Downey et al [22] estimated sample sizes of 325-625 for time to antibiotics after the first evidence of sepsis using the SensiumVitals sensor. A total of 226 participants were randomized; 140 had the sensor applied, and the remaining participants underwent usual care. All outcome measurements in this study demonstrated nonsignificant results. Therefore, we aim to recruit a minimum of 600 individuals, with approximately half in the preimplementation phase and the remaining participants in the postimplementation phase.

### Data Monitoring

Overall, there is a low level of concern for patient safety with the SensiumVitals sensor, given previous use [22]. Furthermore, participants are at very low risk for adverse events; should any occur, they will be logged systematically and reported to ClinicalTrials.gov. Adverse events not related to the sensor will be reported to the responsible clinical team.

## Results

Enrolment is currently underway, having started in September 2017; it is anticipated to end shortly. Data analysis is expected to be completed in 2021.

## Discussion

This trial has the potential to detect earlier early clinical deterioration using the SensiumVitals sensor, which may improve clinical outcomes. This disposable, lightweight, waterproof, wearable wireless patch is attached to a participant’s chest with two adhesive electrocardiogram electrodes and records the participant’s temperature, heart rate, and respiratory rate every 2 minutes, transmitting data to a central monitoring hub through radiofrequency and dedicated intranet hotspots (bridges) installed on wards, viewable through a secured web browser or mobile device. This continuous monitoring enables alerting systems to inform health care staff of individuals whose condition is deteriorating, allowing for earlier intervention.

Acceptability and practicability of continuous monitoring using wearable sensors on general surgical and medical wards has been demonstrated in the United Kingdom and the Netherlands [22,23]. However, these studies focused primarily on feasibility rather than on implementation strategy and clinical outcome measures, such as hospital length of stay, mortality, and intensive care transfers, which remain untested. Furthermore, our use of semistructured interviews to capture stakeholder perceptions will yield pertinent considerations for pragmatic implementation of novel digital technologies.

As a trial designed to test real-world applicability, its design presents inherent limitations. The observational nature of this trial cannot establish cause-effect relationships. However, a prospective evaluation lends itself to describing practical issues that need to be overcome for successful implementation with evolving workflows in health care trusts. Moreover, pre-post designs can be influenced by longitudinal changes in health care delivery, which are a potential source of bias.

In conclusion, the results of our study could offer data to demonstrate the effectiveness of using continuous vital sign monitoring through wearable sensors and digital alerts to improve clinical outcomes in acute general ward settings. We may offer a methodology for successful implementation that can be adopted more widely in various health care trusts.

## Abbreviations

EWS: early warning score
NEWS2: National Early Warning Score 2
